# Supplementary material for: An open label, non-randomized study assessing a prebiotic fiber intervention in a small cohort of Parkinson’s disease participants
Source: Nat Commun. 2023 Feb 18;14:926. doi: 10.1038/s41467-023-36497-x (PMC9938693; doi:10.1038/s41467-023-36497-x)
Supplement: Supplementary file 1 — Supplementary Information [file 41467_2023_36497_MOESM1_ESM.pdf]

Supplementary Information

Supplementary Figure 1

Supplementary Table 1

Supplementary Table 2

Supplementary Table 3

Supplementary Table 4

Supplementary Table 5

Supplementary Table 6

Supplementary Table 7

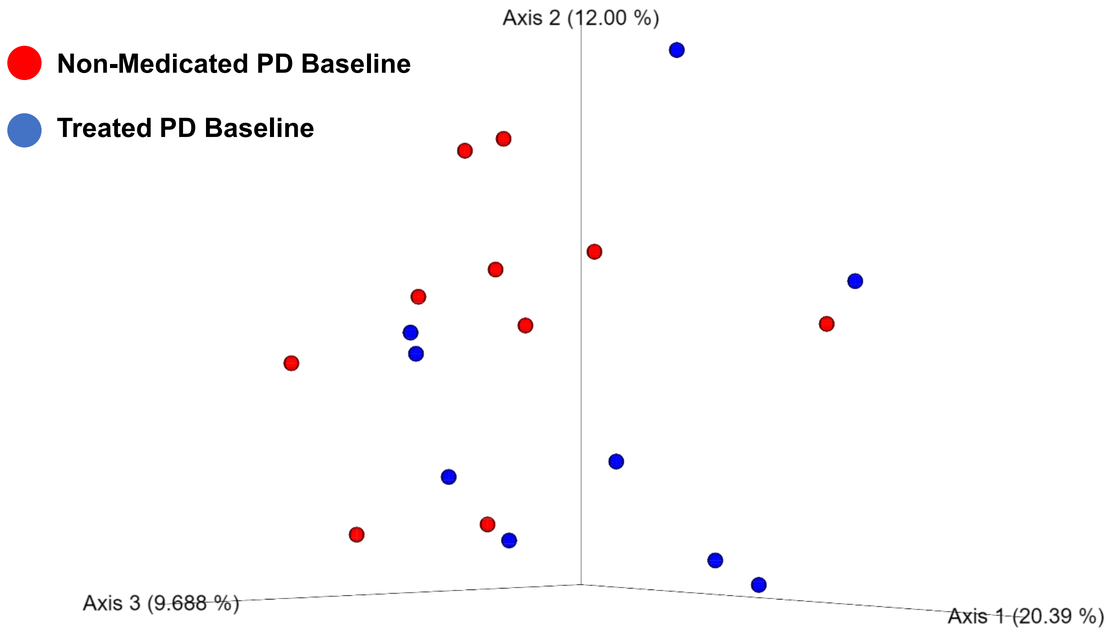

Figure S1. Principal Coordinates Analysis (PCoA) of microbial communities of newly diagnosed, non-medicated (n=10) (red dots) and treated PD participants (n=9) (blue dots) at baseline. Stool microbiota communities were not significantly different between newly diagnosed, non-medicated and treated PD groups at baseline. The PCoA plot was based on Bray-Curtis dissimilarity metrics measured at the taxonomic level of species. PERMDISP: q = 0.85; PERMANOVA: q = 0.28.

Supplementary Table 1. Production of SCFA following stool fermentation.				
	Mean 1 (SD)	Mean 2 (SD)	Mean difference	q-value
Total SCFA (acetate + butyrate + propionate) (mM / 50mg carbohydrate)				
Blank vs. resistant starch	21.04 (0.24)	88.83 (4.96)	-67.79	<0.0001*
Blank vs. rice bran	21.04 (0.24)	49.34 (5.95)	-28.30	0.0003*
Blank vs. resistant maltodextrin	21.04 (0.24)	140.00 (2.67)	-119.0	<0.0001*
Blank vs. inulin	21.04 (0.24)	127.2 (7.36)	-106.2	<0.0001*
Resistant starch vs. rice bran	88.83 (4.96)	49.34 (5.95)	39.50	<0.0001*
Resistant starch vs. resistant maltodextrin	88.83 (4.96)	140.00 (2.67)	-51.16	<0.0001*
Resistant starch vs. inulin	88.83 (4.96)	127.20 (7.36)	-38.40	<0.0001*
Rice bran vs. resistant maltodextrin	49.34 (5.95)	140.00 (2.67)	-90.66	<0.0001*
Rice bran vs. inulin	49.34 (5.95)	127.20 (7.36)	-77.89	<0.0001*
Resistant maltodextrin vs. inulin	140.00 (2.67)	127.20 (7.36)	12.76	0.0601
Acetate (mM / 50mg carbohydrate)				
Blank vs. resistant starch	14.97 (0.06)	34.66 (2.18)	-19.69	<0.0001*
Blank vs. rice bran	14.97 (0.06)	24.91 (2.97)	-9.937	0.0025*
Blank vs. resistant maltodextrin	14.97 (0.06)	66.11 (1.50)	-51.14	<0.0001*
Blank vs. inulin	14.97 (0.06)	61.18 (3.25)	-46.21	<0.0001*
Resistant starch vs. rice bran	34.66 (2.18)	24.91 (2.97)	9.757	0.0028*
Resistant starch vs. resistant maltodextrin	34.66 (2.18)	66.11 (1.50)	-31.45	<0.0001*
Resistant starch vs. inulin	34.66 (2.18)	61.18 (3.25)	-26.51	<0.0001*
Rice bran vs. resistant maltodextrin	24.91 (2.97)	66.11 (1.50)	-41.20	<0.0001*
Rice bran vs. inulin	24.91 (2.97)	61.18 (3.25)	-36.27	<0.0001*
Resistant maltodextrin vs. inulin	66.11 (1.50)	61.18 (3.25)	4.933	0.1373
Butyrate (mM / 50mg carbohydrate)				
Blank vs. resistant starch	3.09 (0.10)	36.16 (1.80)	-33.07	<0.0001*
Blank vs. rice bran	3.09 (0.10)	11.36 (1.41)	-8.270	<0.0001*
Blank vs. resistant maltodextrin	3.09 (0.10)	25.14 (0.52)	-22.05	<0.0001*
Blank vs. inulin	3.09 (0.10)	31.74 (1.62)	-28.65	<0.0001*
Resistant starch vs. rice bran	36.16 (1.80)	11.36 (1.41)	24.80	<0.0001*
Resistant starch vs. resistant maltodextrin	36.16 (1.80)	25.14 (0.52)	11.02	<0.0001*
Resistant starch vs. inulin	36.16 (1.80)	31.74 (1.62)	4.420	0.0116*
Rice bran vs. resistant maltodextrin	11.36 (1.41)	25.14 (0.52)	-13.78	<0.0001*
Rice bran vs. inulin	11.36 (1.41)	31.74 (1.62)	-20.38	<0.0001*
Resistant maltodextrin vs. inulin	25.14 (0.52)	31.74 (1.62)	-6.597	0.0006*
Propionate (mM / 50mg carbohydrate)				
Blank vs. resistant starch	2.98 (0.08)	18.01 (0.98)	-15.03	<0.0001*
Blank vs. rice bran	2.98 (0.08)	13.07 (1.56)	-10.09	<0.0001*
Blank vs. resistant maltodextrin	2.98 (0.08)	48.74 (0.66)	-45.76	<0.0001*
Blank vs. inulin	2.98 (0.08)	34.31 (2.49)	-31.33	<0.0001*
Resistant starch vs. rice bran	18.01 (0.98)	13.07 (1.56)	4.940	0.0112*
Resistant starch vs. resistant maltodextrin	18.01 (0.98)	48.74 (0.66)	-30.73	<0.0001*
Resistant starch vs. inulin	18.01 (0.98)	34.31 (2.49)	-16.30	<0.0001*
Rice bran vs. resistant maltodextrin	13.07 (1.56)	48.74 (0.66)	-35.67	<0.0001*
Rice bran vs. inulin	13.07 (1.56)	34.31 (2.49)	-21.24	<0.0001*
Resistant maltodextrin vs. inulin	48.74 (0.66)	34.31 (2.49)	14.43	<0.0001*

Total SCFA production after stool fermentation. Means derived from triplicate experiments, expressed in mM. SD, standard deviation; vs, versus. Data were analyzed using a one-way ANOVA followed by Tukey's post-hoc test and p-values were adjusted for multiple comparisons which are represented as q-values. *Significance: q-value < 0.05.

Supplementary Table 2: Tolerability of prebiotic bar consumption.

	Post Study Survey (n=20)
Like the bar, n (%)	
Dislike (0-2)	0 (0%)
Somewhat Dislike (3-4)	4 (20%)
Neutral (5-6)	9 (45%)
Somewhat Like (7-8)	4 (20%)
Like (9-10)	3 (15%)
Satisfied with the taste of the bar, n (%)	
Dissatisfied (0-2)	1 (5%)
Somewhat Dissatisfied (3-4)	1 (5%)
Neutral (5-6)	10 (50%)
Somewhat Satisfied (7-8)	5 (25%)
Satisfied (9-10)	3 (15%)
Satisfied with the texture of the bar, n (%)	
Dissatisfied (0-2)	2 (10%)
Somewhat Dissatisfied (3-4)	4 (20%)
Neutral (5-6)	5 (25%)
Somewhat Satisfied (7-8)	6 (30%)
Satisfied (9-10)	3 (15%)
Satisfied with the portion of the bar, n (%)	
Dissatisfied (0-2)	1 (5%)
Somewhat Dissatisfied (3-4)	1 (5%)
Neutral (5-6)	2 (10%)
Somewhat Satisfied (7-8)	7 (35%)
Satisfied (9-10)	9 (45%)
Decreased appetite, n (%)	
Minimal Decrease (0-3)	5 (25%)
Slight Decrease (3-4)	2 (10%)
Moderate Decrease (5-6)	6 (30%)
Decrease (7-8)	4 (20%)
Dramatic Decrease (9-10)	3 (15%)
Want more, n (%)	
Unlikely (0-2)	12 (60%)
Somewhat Unlikely (3-4)	3 (15%)
Somewhat Likely (5-6)	2 (10%)
Likely (7-8)	3 (15%)
Highly Likely (9-10)	0 (0%)
Ease of eating 1 bar, n (%)	
Difficult (0-2)	0 (0%)
Somewhat Difficult (3-4)	0 (0%)
Neutral (5-6)	3 (15%)
Somewhat Easy (7-8)	3 (15%)
Easy (9-10)	14 (70%)
Ease of eating 2 bars, n (%)	
Difficult (0-2)	1 (5%)
Somewhat Difficult (3-4)	2 (10%)
Neutral (5-6)	2 (10%)
Somewhat Easy (7-8)	9 (45%)
Easy (9-10)	6 (30%)
Ease of eating 3 bars, n (%)	
Difficult (0-2)	5 (25%)
Somewhat Difficult (3-4)	4 (20%)
Neutral (5-6)	6 (30%)
Somewhat Easy (7-8)	2 (10%)
Easy (9-10)	3 (15%)
Would continue the bar, n (%)	
Unlikely (0-2)	0 (0%)
Somewhat Unlikely (3-4)	0 (0%)
Somewhat Likely (5-6)	7 (35%)
Likely (7-8)	2 (10%)
Highly Likely (9-10)	11 (55%)

Tolerability questionnaire was a Likert scale: 0-10. Data are shown as n (%). n=20 All PD participants.

Supplementary Table 3. Stool microbial community structure before and after the prebiotic intervention.

PERMANOVA	Pseudo-F	q-value
Baseline: Newly diagnosed, non-medicated PD vs. Treated PD	1.17	0.280
After Prebiotic Treatment: Newly diagnosed, non-medicated PD vs. Treated PD	1.04	0.356
All PD: Baseline vs. Prebiotic	1.00	0.425
Non-Mediated, Newly Diagnosed PD: Baseline vs. Prebiotic	0.75	0.753
Treated PD: Baseline vs. Prebiotic	0.42	0.954
**PERMDISP**	**F-value**	**q-value**
Baseline: Newly diagnosed, non-medicated PD vs. Treated PD	0.031	0.848
After Prebiotic Treatment: Newly diagnosed, non-medicated PD vs. Treated PD	0.318	0.545
All PD: Baseline vs. Prebiotic	3.88	0.051
Newly diagnosed, non-medicated PD: Baseline vs. Prebiotic	1.65	0.192
Treated PD: Baseline vs. Prebiotic	0.53	0.433

Permutational Multivariate Analyses of Variance (PERMANOVA) and Permutational Analyses of Multivariate Dispersions (PERMDISP) were used to analyze microbiota communities. PERMANOVA and PERMDISP calculations were performed on Bray-Curtis distance metrics generated at the taxonomic level of species. Values were determined using 9,999 permutations and adjusted for multiple comparisons using the Benjamini-Hochberg method (q-value). *Significance, q-value < 0.05. n=19 All PD; n=10 newly diagnosed, non-medicated PD; n=9 treated PD.

Supplementary Table 4. Relative abundance of bacterial taxa before and after the prebiotic intervention.				
Taxonomic Level	Baseline Mean RA % ± SD	Prebiotic Mean RA % ± SD	p-value	q-value
Phylum				
Firmicutes	51.08 ± (20.93)	47.09 ± (18.48)	0.395	0.523
Actinobacteria	21.29 ± (18.97)	25.21 ± (21.13)	0.293	0.523
Bacteroidetes	21.27 ± (17.40)	23.79 ± (14.89)	0.418	0.523
Verrucomicrobia	2.71 ± (4.65)	3.01 ± (6.01)	0.783	0.784
Proteobacteria	3.63 ± (5.65)	0.86 ± (1.58)	0.003*	0.016*
Phylum: *Species*				
Actinobacteria: *Bifidobacterium adolescentis*	7.28 ± (12.19)	14.63 ± (17.03)	0.014*	0.212
Firmicutes: *Faecalibacterium prausnitzii*	5.72 ± (5.17)	8.07 ± (6.22)	0.049*	0.313
Bacteroidetes: *Bacteroides uniformis*	5.42 ± (7.26)	5.65 ± (5.70)	0.896	1.000
Actinobacteria: *Collinsella aerofaciens*	5.20 ± (3.67)	5.05 ± (4.15)	0.930	1.000
Firmicutes: *Ruminococcus bromii*	5.38 ± (7.78)	3.25 ± (4.23)	0.014*	0.212
Firmicutes: *Anaerostipes hadrus*	3.78 ± (4.06)	3.43 ± (2.28)	0.921	1.000
Firmicutes: *Fusicatenibacter saccharivorans*	1.65 ± (1.65)	4.61 ± (4.45)	0.001*	0.021*
Firmicutes: *Eubacterium rectale*	2.50 ± (2.71)	3.69 ± (5.30)	0.410	0.816
Verrucomicrobia: *Akkermansia muciniphila*	2.71 ± (4.65)	3.01 ± (6.01)	0.783	1.000
Bacteroidetes: *Alistipes putredinis*	2.28 ± (2.71)	1.80 ± (2.25)	0.530	0.821
Firmicutes: *Roseburia faecis*	2.03 ± (3.32)	1.98 ± (3.76)	0.949	1.000
Actinobacteria: *Bifidobacterium longum*	2.23 ± (3.65)	1.76 ± (2.94)	0.484	0.816
Firmicutes: *Eubacterium hallii*	2.18 ± (2.69)	1.79 ± (1.59)	0.887	1.000
Proteobacteria: *Escherichia coli*	3.29 ± (5.72)	0.58 ± (1.56)	0.029*	0.258
Actinobacteria: *Bifidobacterium pseudocatenulatum*	2.14 ± (3.31)	1.48 ± (3.29)	0.155	0.577
Firmicutes: *Dorea longicatena*	1.63 ± (1.70)	1.88 ± (1.53)	0.206	0.658
Firmicutes: *Blautia obeum*	1.75 ± (2.58)	1.46 ± (1.89)	0.930	1.000
Bacteroidetes: *Alistipes finegoldii*	1.03 ± (1.98)	1.88 ± (2.79)	0.185	0.633
Bacteroidetes: *Prevotella copri*	0.92 ± (2.54)	1.81 ± (7.45)	1.000	1.000
Firmicutes: *Ruminococcus bicirculans*	0.79 ± (1.34)	1.91 ± (2.37)	0.007*	0.212
Bacteroidetes: *Bacteroides vulgatus*	1.25 ± (1.77)	1.32 ± (1.80)	0.443	0.816
Firmicutes: *Ruminococcus torques*	1.64 ± (2.16)	0.70 ± (0.84)	0.021*	0.218
Firmicutes: *Eubacterium siraeum*	1.09 ± (4.11)	1.22 ± (2.93)	0.069	0.411
Firmicutes: *Coprococcus comes*	1.23 ± (1.54)	1.08 ± (1.53)	0.513	0.816
Firmicutes: *Dialister sp.CAG.357*	1.33 ± (3.38)	0.95 ± (2.35)	1.000	0.632
Bacteroidetes: *Parabacteroides merdae*	0.45 ± (0.91)	1.82 ± (3.28)	0.003*	0.044*
Actinobacteria: *Bifidobacterium bifidum*	1.16 ± (3.33)	0.88 ± (2.54)	1.000	1.000

Differences in the relative abundance of individual taxa (relative abundance > 0.1%) before and after the prebiotic intervention were assessed. Mean RA % = average number of sequences per taxa, calculated from the total sum of all sequence counts, depicted as a percentage. SD = standard deviation as a percentage. A two-tailed, Wilcoxon-signed rank test was used to generate p-values and were corrected for multiple comparisons using the Benjamini-Hochberg method (q-value). *Significance: p-value < 0.05; q-value < 0.05. n=19 All PD participants.

Supplementary Table 5. Alpha diversity indices and relative abundance of bacterial taxa alterations before and after the prebiotic intervention by treatment status.				
Taxonomic Level	Baseline (SD)	Prebiotic (SD)	p-value	q-value
Newly diagnosed, non-medicated PD Participants				
Diversity Index				
Shannon Index	3.10 ± (0.30)	3.01 ± (0.24)	0.217	-
Simpson	0.92 ± (0.03)	0.90 ± (0.05)	0.108	-
Species Richness	77.50 ± (7.66)	74.80 ± (11.75)	0.326	-
Evenness	0.71 ± (0.06)	0.70 ± (0.05)	0.383	-
Phylum				
Firmicutes	60.39 ± (18.15)	53.69 ± (16.58)	0.322	0.615
Actinobacteria	16.24 ± (19.92)	19.23 ± (16.45)	0.492	0.615
Bacteroidetes	18.58 ± (16.77)	23.96 ± (13.00)	0.431	0.615
Verrucomicrobia	1.90 ± (2.96)	2.21 ± (3.82)	1.000	1.000
Proteobacteria	2.87 ± (4.96)	0.91 ± (2.16)	0.083	0.420
Phylum: *Species*				
Actinobacteria: *Bifidobacterium adolescentis*	3.43 ± (10.28)	10.36 ± (15.79)	0.100	0.562
Firmicutes: *Faecalibacterium prausnitzii*	6.84 ± (6.30)	9.55 ± (6.76)	0.064	0.478
Bacteroidetes: *Bacteroides uniformis*	4.11 ± (5.28)	5.78 ± (5.40)	0.375	0.856
Actinobacteria: *Collinsella aerofaciens*	6.07 ± (3.50)	5.62 ± (3.42)	1.000	1.000
Firmicutes: *Ruminococcus bromii*	6.92 ± (9.84)	3.99 ± (4.79)	0.105	0.562
Firmicutes: *Anaerostipes hadrus*	3.05 ± (3.57)	3.11 ± (2.33)	0.769	1.000
Firmicutes: *Fusicatenibacter saccharivorans*	1.89 ± (1.29)	5.20 ± (4.86)	0.009*	0.317
Firmicutes: *Eubacterium rectale*	2.46 ± (2.77)	2.72 ± (3.09)	0.726	1.000
Verrucomicrobia: *Akkermansia muciniphila*	1.90 ± (2.96)	2.21 ± (3.82)	1.000	1.000
Bacteroidetes: *Alistipes putredinis*	3.23 ± (2.80)	2.02 ± (1.58)	0.192	0.578
Firmicutes: *Roseburia faecis*	2.57 ± (4.07)	2.61 ± (4.27)	0.528	0.981
Actinobacteria: *Bifidobacterium longum*	2.25 ± (4.57)	0.97 ± (1.29)	0.529	0.981
Firmicutes: *Eubacterium hallii*	2.21 ± (3.48)	1.65 ± (1.23)	1.000	1.000
Proteobacteria: *Escherichia coli*	2.67 ± (4.96)	0.81 ± (2.16)	0.141	0.578
Actinobacteria: *Bifidobacterium pseudocatenulatum*	1.25 ± (2.61)	0.45 ± (0.94)	0.201	0.578
Firmicutes: *Dorea longicatena*	2.45 ± (1.92)	2.62 ± (1.41)	0.695	1.000
Firmicutes: *Blautia obeum*	2.07 ± (1.47)	2.21 ± (2.33)	1.000	1.000
Bacteroidetes: *Alistipes finegoldii*	0.65 ± (1.62)	0.97 ± (1.42)	0.624	1.000
Bacteroidetes: *Prevotella copri*	1.61 ± (3.40)	3.44 ± (10.24)	1.000	1.000
Firmicutes: *Ruminococcus bicirculans*	1.25 ± (1.80)	2.94 ± (2.66)	0.014*	0.317
Bacteroidetes: *Bacteroides vulgatus*	0.99 ± (1.55)	1.05 ± (1.42)	0.183	0.578
Firmicutes: *Ruminococcus torques*	2.31 ± (2.43)	0.81 ± (0.84)	0.024*	0.362
Firmicutes: *Eubacterium siraeum*	0.21 ± (0.28)	0.80 ± (1.02)	0.042*	0.471
Firmicutes: *Coprococcus comes*	1.54 ± (1.96)	1.21 ± (1.96)	0.769	1.000
Firmicutes: *Dialister sp.CAG.357*	1.23 ± (3.91)	0.92 ± (2.92)	1.000	1.000
Bacteroidetes: *Parabacteroides merdae*	0.45 ± (0.63)	1.79 ± (2.16)	0.014*	0.317
Actinobacteria: *Bifidobacterium bifidum*	0.77 ± (2.38)	0.23 ± (0.55)	1.000	1.000
Treated PD Participants				
Diversity Index				
Shannon Index	2.96 ± (0.41)	2.76 ± (0.35)	0.134	-
Simpson	0.90 ± (0.05)	0.87 ± (0.07)	0.233	-
Species Richness	71.78 ± (12.02)	69.44 ± (9.92)	0.424	-
Evenness	0.69 ± (0.08)	0.65 ± (0.09)	0.214	-
Phylum				
Firmicutes	40.73 ± (19.65)	39.76 ± (18.55)	0.910	0.932
Actinobacteria	26.90 ± (17.20)	31.87 ± (24.60)	0.496	0.932
Bacteroidetes	24.25 ± (18.60)	23.64 ± (17.57)	0.910	0.932
Verrucomicrobia	3.60 ± (6.09)	3.90 ± (7.95)	0.932	0.932
Proteobacteria	4.48 ± (6.53)	0.80 ± (0.59)	0.027*	0.137
Phylum: *Species*				
Actinobacteria: *Bifidobacterium adolescentis*	11.56 ± (13.28)	19.38 ± (18.00)	0.108	0.978
Firmicutes: *Faecalibacterium prausnitzii*	4.47 ± (3.50)	6.42 ± (5.45)	0.496	1.000
Bacteroidetes: *Bacteroides uniformis*	6.88 ± (9.09)	5.05 ± (6.34)	0.441	1.000
Actinobacteria: *Collinsella aerofaciens*	4.24 ± (3.81)	4.42 ± (4.98)	0.833	1.000
Firmicutes: *Ruminococcus bromii*	3.67 ± (4.59)	2.42 ± (3.59)	0.105	0.978

Firmicutes: *Anaerostipes hadrus*	4.58 ± (4.62)	3.79 ± (2.31)	0.734	1.000
Firmicutes: *Fusicatenibacter saccharivorans*	1.37 ± (2.03)	3.95 ± (4.13)	0.021*	0.978
Firmicutes: *Eubacterium rectale*	2.55 ± (2.81)	4.77 ± (7.07)	0.446	1.000
Verrucomicrobia: *Akkermansia muciniphila*	3.60 ± (6.09)	3.90 ± (7.95)	0.932	1.000
Bacteroidetes: *Alistipes putredinis*	1.23 ± (2.31)	1.56 ± (2.92)	0.418	1.000
Firmicutes: *Roseburia faecis*	1.43 ± (2.31)	1.28 ± (3.20)	0.529	1.000
Actinobacteria: *Bifidobacterium longum*	2.22 ± (2.54)	2.65 ± (3.99)	0.799	1.000
Firmicutes: *Eubacterium hallii*	2.15 ± (1.63)	1.95 ± (1.99)	0.944	1.000
Proteobacteria: *Escherichia coli*	3.97 ± (5.72)	0.33 ± (0.38)	0.183	0.978
Actinobacteria: *Bifidobacterium pseudocatenulatum*	3.12 ± (3.87)	2.64 ± (4.53)	0.589	1.000
Firmicutes: *Dorea longicatena*	0.71 ± (0.75)	1.07 ± (1.28)	0.799	1.000
Firmicutes: *Blautia obeum*	1.38 ± (3.51)	0.63 ± (0.68)	0.820	1.000
Bacteroidetes: *Alistipes finegoldii*	1.45 ± (2.33)	2.90 ± (3.62)	0.203	0.978
Bacteroidetes: *Prevotella copri*	0.16 ± (0.49)	0.003 ± (0.009)	1.000	1.000
Firmicutes: *Ruminococcus bicirculans*	0.28 ± (0.26)	0.76 ± (1.35)	0.544	1.000
Bacteroidetes: *Bacteroides vulgatus*	1.53 ± (2.04)	1.62 ± (2.21)	0.932	1.000
Firmicutes: *Ruminococcus torques*	0.89 ± (1.62)	0.58 ± (0.88)	0.799	1.000
Firmicutes: *Eubacterium siraeum*	2.07 ± (5.99)	1.69 ± (4.21)	0.589	1.000
Firmicutes: *Coprococcus comes*	0.88 ± (0.88)	0.93 ± (0.95)	0.441	1.000
Firmicutes: *Dialister sp.CAG.357*	1.43 ± (2.92)	0.98 ± (1.68)	0.361	0.978
Bacteroidetes: *Parabacteroides merdae*	0.46 ± (1.20)	1.85 ± (4.35)	0.201	0.978
Actinobacteria: *Bifidobacterium bifidum*	1.60 ± (4.27)	1.61 ± (3.62)	1.000	1.000

Shannon Index, Simpson's Index, Species Richness and Pielou's evenness were measured at the taxonomic level of species. Differences in the relative abundance of individual taxa (relative abundance > 0.1%) before and after the prebiotic intervention were assessed. Mean RA % = average number of sequences per taxa, calculated from the total sum of all sequence counts, depicted as a percentage. SD = standard deviation as a percentage. A two-tailed, Wilcoxon-signed rank test was used to generate p-values and were corrected for multiple comparisons using the Benjamini-Hochberg method (q-value). *Significance: p-value < 0.05; q-value < 0.05. n=10 newly diagnosed, non-medicated PD; n=9 treated PD.

Supplementary Table 6. Differential abundance of functional gene pathways before and after the prebiotic intervention.					
Significant Functional Gene Pathways	Baseline Mean Abundance	Prebiotic Mean Abundance	Log2FC	p-value	q-value
P164-PWY: purine nucleobases degradation I (anaerobic)	607.65	487.61	-0.32	0.001*	0.017*
TEICHOICACID-PWY: teichoic acid (poly-glycerol) biosynthesis	414.14	320.13	-0.37	0.001*	0.017*
PWY-7210: pyrimidine deoxyribonucleotides biosynthesis from CTP	844.62	615.23	-0.46	0.000*	0.017*
PWY-5188: tetrapyrrole biosynthesis I (from glutamate)	1272.45	921.54	-0.47	0.001*	0.017*
ASPASN-PWY: superpathway of L-aspartate and L-asparagine biosynthesis	1515.34	1043.34	-0.54	0.000*	0.017*
PWY-5104: L-isoleucine biosynthesis IV	1871.58	1276.48	-0.55	0.000*	0.017*
PWY-6608: guanosine nucleotides degradation III	1386.07	884.77	-0.65	0.001*	0.017*
PWY66-409: superpathway of purine nucleotide salvage	996.50	488.10	-1.03	0.001*	0.017*
PWY-4981: L-proline biosynthesis II (from arginine)	684.93	328.69	-1.06	0.001*	0.017*
PWY0-1479: tRNA processing	720.80	281.93	-1.35	0.000*	0.017*
MET-SAM-PWY: superpathway of S-adenosyl-L-methionine biosynthesis	1580.85	1098.93	-0.52	0.001*	0.019*
PWY-7211: superpathway of pyrimidine deoxyribonucleotides de novo biosynthesis	1144.49	879.95	-0.38	0.002*	0.019*
PWY-6545: pyrimidine deoxyribonucleotides de novo biosynthesis III	951.92	729.06	-0.38	0.001*	0.019*
PWY-7220: adenosine deoxyribonucleotides de novo biosynthesis II	2598.75	1932.85	-0.43	0.002*	0.019*
PWY-7222: guanosine deoxyribonucleotides de novo biosynthesis II	2598.75	1932.85	-0.43	0.002*	0.019*
PWY-5347: superpathway of L-methionine biosynthesis (transsulfuration)	1485.71	1022.12	-0.54	0.001*	0.019*
METSYN-PWY: L-homoserine and L-methionine biosynthesis	1492.30	1013.20	-0.56	0.001*	0.019*
HOMOSER-METSYN-PWY: L-methionine biosynthesis I	957.68	619.77	-0.63	0.002*	0.019*
FUC-RHAMCAT-PWY: superpathway of fucose and rhamnose degradation	486.61	281.12	-0.79	0.002*	0.019*
PWY-6628: superpathway of L-phenylalanine biosynthesis	652.12	229.53	-1.51	0.002*	0.019*
PWY-841: superpathway of purine nucleotides de novo biosynthesis I	1839.62	1419.98	-0.37	0.002*	0.021*
PWY-6892: thiazole biosynthesis I (E. coli)	2181.98	1744.11	-0.32	0.002*	0.022*
ARG-POLYAMINE-SYN: superpathway of arginine and polyamine biosynthesis	802.88	477.74	-0.75	0.002*	0.022*
POLYAMSYN-PWY: superpathway of polyamine biosynthesis I	508.71	281.69	-0.85	0.002*	0.022*
PWY-7184: pyrimidine deoxyribonucleotides de novo biosynthesis I	1132.29	798.22	-0.50	0.003*	0.025*
PWY0-166: superpathway of pyrimidine deoxyribonucleotides de novo biosynthesis (E. coli)	1555.16	1202.56	-0.37	0.003*	0.027*
PWY-7208: superpathway of pyrimidine nucleobases salvage	1097.81	821.67	-0.42	0.003*	0.027*
DENOVOPURINE2-PWY: superpathway of purine nucleotides de novo biosynthesis II	1918.89	1520.45	-0.34	0.004*	0.028*
PWY-6125: superpathway of guanosine nucleotides de novo biosynthesis II	1510.98	1117.02	-0.44	0.004*	0.028*
PWY0-1297: superpathway of purine deoxyribonucleosides degradation	770.24	425.16	-0.86	0.004*	0.028*
PWY-7187: pyrimidine deoxyribonucleotides de novo biosynthesis II	1549.82	1216.08	-0.35	0.005*	0.029*
PWY-2941: L-lysine biosynthesis II	940.31	675.77	-0.48	0.005*	0.029*

PWY-5345: superpathway of L-methionine biosynthesis (by sulfhydrylation)	794.65	345.74	-1.20	0.005*	0.029*
SULFATE-CYS-PWY: superpathway of sulfate assimilation and cysteine biosynthesis	745.62	294.23	-1.34	0.005*	0.029*
PWY-7198: pyrimidine deoxyribonucleotides de novo biosynthesis IV	907.03	693.68	-0.39	0.005*	0.030*
PWY-7228: superpathway of guanosine nucleotides de novo biosynthesis I	1420.60	1036.25	-0.46	0.005*	0.030*
PWY4LZ-257: superpathway of fermentation (Chlamydomonas reinhardtii)	580.58	279.26	-1.06	0.005*	0.030*
P161-PWY: acetylene degradation	536.12	242.82	-1.14	0.005*	0.030*
PWY-6126: superpathway of adenosine nucleotides de novo biosynthesis II	3056.38	2491.45	-0.29	0.006*	0.033*
SALVADEHYPOX-PWY: adenosine nucleotides degradation II	1175.87	729.95	-0.69	0.006*	0.033*
GLUCOSE1PMETAB-PWY: glucose and glucose-1-phosphate degradation	522.38	241.36	-1.11	0.006*	0.033*
PWY-1042: glycolysis IV (plant cytosol)	4347.25	3861.78	-0.17	0.008*	0.035*
TRPSYN-PWY: L-tryptophan biosynthesis	2113.63	1806.04	-0.23	0.008*	0.035*
PWY-7229: superpathway of adenosine nucleotides de novo biosynthesis I	3260.36	2705.79	-0.27	0.008*	0.035*
PWY-7197: pyrimidine deoxyribonucleotide phosphorylation	764.77	550.03	-0.48	0.008*	0.035*
HEXITOLDEGSUPER-PWY: superpathway of hexitol degradation (bacteria)	1330.33	924.41	-0.53	0.008*	0.035*
PWY-5676: acetyl-CoA fermentation to butanoate II	607.71	421.37	-0.53	0.007*	0.035*
PWY-6606: guanosine nucleotides degradation II	691.52	405.20	-0.77	0.007*	0.035*
PWY-7328: superpathway of UDP-glucose-derived O-antigen building blocks biosynthesis	508.07	253.94	-1.00	0.007*	0.035*
PWY-6113: superpathway of mycolate biosynthesis	709.66	279.59	-1.34	0.008*	0.035*
GLYCOLYSIS-TCA-GLYOX-BYPASS: superpathway of glycolysis, pyruvate dehydrogenase, TCA, and glyoxylate bypass	586.22	145.15	-2.01	0.008*	0.035*
GLUTORN-PWY: L-ornithine biosynthesis	2920.17	2581.73	-0.18	0.011*	0.044*
PWY66-422: D-galactose degradation V (Leloir pathway)	2483.03	2146.14	-0.21	0.011*	0.044*
PWY-6353: purine nucleotides degradation II (aerobic)	1119.04	723.09	-0.63	0.011*	0.044*
PWY-7111: pyruvate fermentation to isobutanol (engineered)	4542.19	3930.69	-0.21	0.012*	0.045*
PWY-5667: CDP-diacylglycerol biosynthesis I	2794.61	2356.61	-0.25	0.012*	0.045*
PWY0-1319: CDP-diacylglycerol biosynthesis II	2794.61	2356.61	-0.25	0.012*	0.045*
PWY-6703: preQ0 biosynthesis	1585.87	1293.57	-0.29	0.012*	0.045*
PWY0-781: aspartate superpathway	1078.46	737.51	-0.55	0.012*	0.045*
FAO-PWY: fatty acid β-oxidation I	593.82	201.47	-1.56	0.012*	0.045*
PWY0-1586: peptidoglycan maturation (meso-diaminopimelate containing)	2773.53	2142.53	-0.37	0.014*	0.048*
PRPP-PWY: superpathway of histidine, purine, and pyrimidine biosynthesis	1363.95	1037.23	-0.40	0.014*	0.048*
PWY-621: sucrose degradation III (sucrose invertase)	1113.73	787.71	-0.50	0.014*	0.048*
PWY-5971: palmitate biosynthesis II (bacteria & plants)	903.19	441.24	-1.03	0.014*	0.048*

Differences in the relative abundance of functional genes/pathways (relative abundance > 0.1%) before and after the prebiotic intervention were assessed. Log2 fold change calculated for each pathway (baseline and after the intervention). Mean Abundance = average number of sequences. A two-tailed, Wilcoxon-signed rank test was used to identify significantly differentially abundant genes/pathways between matched samples (baseline and the intervention) and p-values were corrected for multiple comparisons using the Benjamini-Hochberg method (q-value). *Significance: p-value < 0.05; q-value < 0.05. n=19 All PD participants.

Supplementary Table 7. Biological outcomes before and after the prebiotic intervention by treatment status.			
	Baseline (SD)	Prebiotic (SD)	p-value
Newly Diagnosed, non-Medicated PD Participants			
Short Chain Fatty Acids - Plasma			
Acetate (ug/mL) (n=9)	1.96 (0.78)	2.45 (0.54)	0.128
Propionate (ug/mL) (n=9)	0.09 (0.07)	0.11 (0.04)	0.250
Butyrate (ug/mL) (n=9)	0.07 (0.04)	0.07 (0.03)	0.570
Total SCFA (ug/mL) (n=9)	2.13 (0.84)	2.63 (0.51)	0.128
Butyrate / Total SCFA Ratio (n=9)	0.03 (0.02)	0.03 (0.01)	0.426
(Propionate + Butyrate) / Total SCFA Ratio (n=9)	0.13 (0.07)	0.13 (0.05)	0.425
Intestinal Barrier Integrity, Bacterial Translocation, Intestinal Inflammation			
Plasma Zonulin (ng/ml)	21.37 (7.66)	14.70 (5.59)	0.088
Plasma LBP (ng/ml)	14,321 (6,131)	16,946 (8,894)	0.094
Stool Calprotectin (ug/g)	120.82 (140.68)	90.35 (113.69)	0.067
Systemic Inflammation - Serum			
IFN-γ (pg/ml)	3.15 (1.57)	3.30 (2.12)	0.556
IL-6 (pg/ml)	0.62 (0.31)	0.76 (0.58)	0.695
IL-8 (pg/ml)	10.43 (2.93)	10.20 (3.53)	0.764
IL-10 (pg/ml)	0.24 (0.11)	0.27 (0.10)	0.084
TNF-α (pg/ml)	0.64 (0.14)	0.67 (0.22)	0.344
CRP (mg/L)	1.50 (1.58)	2.90 (4.33)	0.500
Brain Outcomes – Serum			
BDNF (pg/ml)	5,740 (1,558)	5,572 (1,689)	0.756
NfL (pg/ml) (n=9)	56.07 (24.99)	48.92 (23.62)	0.008*
HMGB-1 (ng/ml)	240.63 (94.73)	200.64 (70.40)	0.299
Treated PD Participants			
Short Chain Fatty Acids - Plasma			
Acetate (ug/mL) (n=9)	1.84 (0.65)	3.01 (1.75)	0.027*
Propionate (ug/mL) (n=9)	0.07 (0.02)	0.13 (0.06)	0.011*
Butyrate (ug/mL) (n=9)	0.06 (0.04)	0.08 (0.04)	0.019*
Total SCFA (ug/mL) (n=9)	1.97 (0.67)	3.22 (1.78)	0.019*
Butyrate / Total SCFA Ratio (n=9)	0.03 (0.02)	0.03 (0.02)	1.000
(Propionate + Butyrate) / Total SCFA Ratio (n=9)	0.10 (0.04)	0.16 (0.06)	0.054
Intestinal Barrier Integrity, Bacterial Translocation, Intestinal Inflammation			
Plasma Zonulin (ng/ml)	20.79 (5.81)	13.24 (4.26)	<0.001*
Plasma LBP (ng/ml)	14,631 (5,996)	13,464 (2,594)	0.845
Stool Calprotectin (ug/g)	28.07 (22.61)	19.56 (11.01)	0.169
Systemic Inflammation - Serum			
IFN-γ (pg/ml)	9.61 (19.73)	3.43 (2.83)	0.275
IL-6 (pg/ml)	0.72 (0.40)	0.64 (0.35)	0.322
IL-8 (pg/ml)	43.02 (106.92)	9.84 (2.82)	0.921
IL-10 (pg/ml)	0.34 (0.29)	0.40 (0.45)	0.921
TNF-α (pg/ml)	0.71 (0.25)	0.69 (0.25)	0.757
CRP (mg/L)	2.90 (6.01)	1.10 (0.32)	0.999
Brain Outcomes – Serum			
BDNF (pg/ml)	5,701 (1,356)	5,108 (1,770)	0.405
NfL (pg/ml)	82.58 (39.22)	75.94 (36.66)	0.091
HMGB-1 (ng/ml)	206.29 (120.25)	218.09 (95.59)	0.603

All data are shown as mean (standard deviation). BDNF, brain derived-neurotrophic-factor; CRP, C-Reactive Protein; HMGB-1, high mobility group box 1 protein; IFN-γ, interferon-gamma; IL, interleukin; LBP, lipopolysaccharide binding protein; NfL, neurofilament light chain; SD, standard deviation; TNF-α, tumor necrosis factor-alpha. Based on the Shapiro-Wilks normality test, either a two-tailed, parametric paired t-test or a two-tailed non-parametric Wilcoxon signed-rank test was used for analysis. *Significance: p-value < 0.05. n=10 newly diagnosed, non-medicated PD; n=10 treated PD, unless noted above.
